# Bus-Mounted Vision Sensing for Traffic Object Detection: BFTD and a Local–Global Attention Framework

**DOI:** 10.3390/s26155001

**Published:** 2026-08-06

**Authors:** Wenjing Gao, Nan Zou

**Affiliations:** 1 School of Software, Shandong University, Jinan 250100, China; gaowenjing0930@gmail.com; 2 School of Control Science and Engineering, Shandong University, Jinan 250014, China

**Keywords:** bus-mounted vision sensing, intelligent transportation systems, local–global attention, object detection, small object detection, traffic scene perception

## Abstract

Bus-mounted vision sensing provides a practical and complementary perspective for intelligent transportation systems, but reliable traffic object detection from bus front-view cameras remains challenging because elevated viewpoints induce severe scale skewness, dense interactions around bus stops and intersections, and frequent heterogeneous occlusion. To support this sensing scenario while avoiding ambiguity with previously used dataset acronyms, we construct the Bus Front-view Traffic Dataset (BFTD), a high-resolution benchmark collected from forward-facing cameras mounted on multiple buses operating on urban routes during real-world service. The BFTD contains 8131 images and 56,137 annotated instances across five traffic-participant categories, covering dense pedestrians, mixed-traffic flow, illumination variation, rain, fog, and occlusion-prone scenes. Based on the visual characteristics of bus-mounted cameras, we propose YOLO-M2LA, a local–global attention detection framework in which CBS-SPD preserves fine-grained information during early downsampling and M2LA couples multi-scale local context modeling with efficient global dependency aggregation. Extensive experiments on BFTD and public benchmarks show that the proposed framework improves detection accuracy, particularly for small and visually crowded traffic participants, while maintaining a practical accuracy–efficiency trade-off. Dataset statistics, condition-specific evaluation, ablation analysis, and qualitative visualization further support the effectiveness of BFTD and YOLO-M2LA for vision-based traffic sensing. The dataset and implementation are publicly available online.

## 1. Introduction

Object detection is a core task in vision-based sensing and provides essential support for practical scene perception in real-world environments [1,2]. In traffic-related applications, reliable detection is required for understanding complex visual scenes containing pedestrians, cyclists, and vehicles under varying viewpoints, densities, and environmental conditions. Despite substantial advances in deep learning, robust detection remains difficult when targets are small, densely distributed, or partially occluded. These challenges are particularly evident in bus-mounted front-view videos, where the elevated viewpoint causes pronounced scale variation with depth, and mixed-traffic interactions around bus stops and intersections introduce frequent visual ambiguity.

Recent advances in deep learning have considerably improved detection accuracy and efficiency through stronger feature representation and context modeling. Representative two-stage detectors such as Faster R-CNN [3] and Cascade R-CNN [4] achieve strong localization performance through region proposal mechanisms, while one-stage detectors, including SSD [5], RetinaNet [6], and the YOLO family [7,8,9], offer competitive accuracy with efficient inference. Transformer-based detectors such as Deformable DETR [10] and DINO [11] further strengthen global reasoning and multi-scale representation. In parallel, feature pyramid designs [12] and attention-based mechanisms [13,14] have been widely adopted to improve detection under small-object and occlusion-prone conditions, where limited spatial resolution and weak discriminative cues remain major sources of performance degradation [15,16].

**Figure 1:**
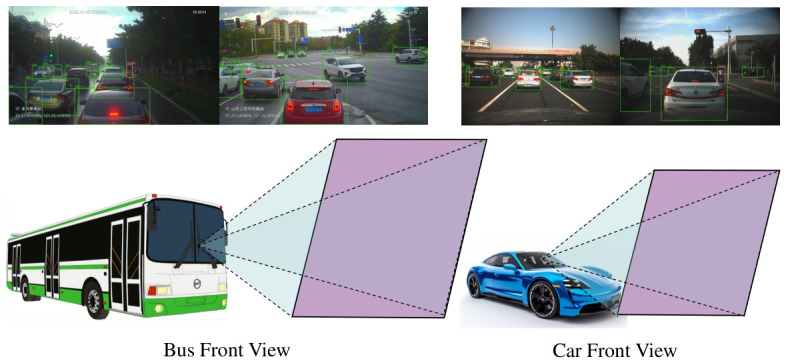
Comparison between bus and car front-view perspectives. A higher camera installation on buses typically provides a broader field of view and shifts the scale distribution toward smaller vulnerable road users at distance, while crowded bus stops and intersections still induce frequent dynamic occlusions.

Although these methods have shown strong performance on mainstream benchmarks, most existing traffic detection datasets are developed primarily from the private-car perspective. As a result, they do not fully reflect the visual characteristics of bus-mounted front-view scenes, where viewpoint geometry, scale distribution, and traffic composition differ substantially from those in conventional low-view front-view perception. As illustrated in Figure 1, the elevated camera viewpoint on buses enlarges the observable traffic region but also produces a more challenging visual structure: vulnerable road users such as pedestrians and cyclists frequently appear as very small targets in distant regions, whereas nearby vehicles occupy medium- and large-scale areas. At the same time, mixed-traffic interactions around bus stops and intersections often generate frequent and heterogeneous occlusions among pedestrians, cyclists, and vehicles. These properties make bus front-view scenes a distinct and practically important object detection setting.

From the perspective of visual modeling, the difficulty of bus front-view detection can be characterized by two coupled structural challenges. The first is severe scale skewness induced by the elevated viewpoint. In such scenes, small vulnerable road users are densely distributed in distant regions, while larger vehicles dominate nearby areas. Conventional early downsampling operations may therefore discard fine-grained spatial cues that are crucial for detecting small and distant objects [15]. The second challenge is heterogeneous dynamic occlusion in mixed-traffic scenes. In crowded bus-stop and intersection environments, reliable recognition often depends not only on local appearance details but also on broader contextual reasoning to disambiguate overlapping or partially visible targets [16]. Existing generic detection architectures do not explicitly address this combination of extreme scale imbalance and occlusion-rich scene composition.

These observations indicate the need for both a dedicated benchmark that reflects the visual characteristics of real bus-mounted front-view videos and a detection framework designed for this practical machine vision setting. To this end, we construct the Bus Front-view Traffic Dataset (BFTD), a high-resolution benchmark collected from forward-facing cameras mounted on multiple buses operating on multiple urban routes. BFTD captures dense pedestrian regions, mixed-traffic flow, frequent occlusions, and diverse environmental conditions observed in real bus front-view scenes. Based on the two structural challenges identified above, we further propose YOLO-M2LA, a mechanism-oriented detection framework for bus front-view object detection. In this framework, CBS-SPD is used to preserve fine-grained cues during early downsampling, while M2LA integrates multi-scale local context modeling with efficient global dependency aggregation to improve robust target disambiguation. Together, BFTD and YOLO-M2LA provide a practical benchmark-and-method framework for bus-mounted visual sensing in urban traffic perception.

From an engineering perspective, bus-mounted visual perception is closely related to intelligent transportation systems, public transportation safety, and urban traffic monitoring. Different from private-car-oriented autonomous driving datasets, bus front-view scenes provide a complementary sensing perspective for traffic participants around bus stops, intersections, and mixed-flow road segments. Therefore, developing a reproducible benchmark and an efficient detector for this scenario is valuable for practical vision-based transportation perception.

The main contributions of this work are summarized as follows:

- We construct BFTD, a dedicated high-resolution benchmark for bus-mounted front-view traffic sensing. BFTD captures the real object composition and visual complexity of elevated-view urban traffic scenes, including dense pedestrian regions, heterogeneous traffic flows, adverse visual conditions, and frequent occlusions.
- We provide a scene-specific analysis of bus front-view perception, including category composition, environmental conditions, and annotation-derived visual statistics. This analysis highlights the coupled challenges of scale skewness, object density, and occlusion-prone mixed traffic.
- We propose YOLO-M2LA, a local–global attention detection framework in which CBS-SPD preserves fine-grained spatial information during early downsampling and M2LA enhances multi-scale local reasoning and efficient global contextual aggregation for occlusion-prone traffic scenes.
- Extensive experiments, including overall comparison, condition-specific evaluation, component ablation, cross-dataset transfer, and qualitative visualization, demonstrate that the proposed framework improves small-object and crowded-scene detection while maintaining a practical accuracy–efficiency trade-off for vision-based traffic sensing.

## 2. Related Work

### 2.1. Small and Occluded Object Detection

Small-object detection remains a long-standing challenge in computer vision because small targets are represented by limited pixels, weak semantic cues, and insufficient discriminative details [15]. These difficulties are often amplified by clutter, scale variation, and partial occlusion, especially in forward-looking urban scenes. A major line of research addresses this problem through multi-scale representation learning. The Feature Pyramid Network (FPN) [12] established the foundation for hierarchical feature fusion, and many later studies further improved small-object representation through stronger multi-scale aggregation and feature interaction. For example, Qu et al. [17] proposed an attention-based multi-scale fusion strategy, while Quan et al. [18] introduced the Centralized Feature Pyramid to combine global dependencies with local discriminative cues.

In addition to multi-scale representation, contextual reasoning plays an important role in detecting small and partially occluded targets. Since the visual evidence of small objects is often incomplete, surrounding spatial context can provide essential support for recognition and localization. Hu et al. [13] proposed SINet with context-aware pooling for small-object detection, and Luo et al. [14] developed a multi-scale context enhancement network to improve perception in complex scenes. More generally, occlusion handling has been recognized as a key issue in generic object detection [16], particularly when overlapping objects become difficult to separate from one another or from the background.

Despite these advances, most existing approaches are evaluated on general-purpose benchmarks or conventional vehicle-mounted datasets. Such settings do not fully capture the combination of severe scale skewness and heterogeneous dynamic occlusion that appears in bus front-view scenes. As a result, bus front-view detection forms a representative scene-specific instance of small and occluded object detection, where both fine-grained feature preservation and robust contextual disambiguation are particularly important.

Recent transportation-oriented studies further show that object detection performance is strongly affected by traffic density, occlusion, and environmental variation. For example, Bakirci investigated YOLOv8 variants for vehicle classification and intelligent transportation monitoring under aerial sensing conditions, providing useful benchmarking context for transportation-oriented detector evaluation [19]. Different from these aerial or roadside sensing settings, BFTD focuses on bus-mounted front-view perception, where the camera is mobile, elevated, and directly embedded in public transportation operation.

### 2.2. Context Modeling for Visually Challenging Detection Scenes

Context modeling has become a central component in modern object detection. Classical CNN-based detectors such as Faster R-CNN [3], RetinaNet [6], and the YOLO family [7,8,9] have achieved strong performance by combining effective feature extraction with efficient inference. More recently, transformer-based detectors, including Deformable DETR [10], DINO [11], and F-DETR [20], have further demonstrated the value of global dependency modeling and multi-scale reasoning.

To reduce the limitations of local receptive fields while retaining computational efficiency, many studies have explored lightweight context modeling strategies. In CNN-based designs, contextual information is often enhanced through enlarged receptive fields, dilated operations, and cross-scale feature interaction. In attention-based designs, local attention and efficient global aggregation are increasingly used to approximate the benefits of full self-attention with lower cost. These developments are particularly relevant for visually challenging detection scenes, where local appearance alone is insufficient and broader scene-level relationships are required for reliable target disambiguation.

Nevertheless, existing context modeling strategies are typically developed for general detection settings and are not explicitly designed for the coupled challenges found in bus front-view scenes. In such scenes, early feature degradation caused by downsampling interacts with contextual ambiguity caused by dense mixed traffic and frequent occlusion. This observation motivates the mechanism-oriented design adopted in this work, where CBS-SPD focuses on preserving fine-grained cues under severe scale skewness and M2LA enhances local–global reasoning for occlusion-aware disambiguation.

### 2.3. Vision-Based Traffic Datasets and Benchmark Gaps

Public benchmarks have played a central role in advancing object detection and traffic scene understanding. Representative datasets such as KITTI [21], Cityscapes [22], BDD100K [23], nuScenes [24], Waymo Open [25], ApolloScape [26], and Argoverse [27] have significantly promoted research on autonomous driving perception. Other datasets, including Caltech Pedestrian [28], CrowdHuman [29], VisDrone [30], and UA-DETRAC [31], provide valuable benchmarks for pedestrian detection, crowd analysis, aerial small-object detection, and roadside surveillance. These datasets cover a wide range of viewpoints and tasks, but their visual properties differ substantially from those of bus-mounted front-view scenes.

As summarized in Table 1, most mainstream traffic benchmarks are built from private-car viewpoints, aerial platforms, or static roadside cameras. Consequently, they do not fully reflect the elevated viewpoint, extreme depth-induced scale variation, and mixed-traffic occlusion patterns observed in bus front-view videos. Existing studies have shown that cameras mounted on transit buses can support practical traffic monitoring and scene analysis [32], suggesting that the bus-mounted viewpoint is both meaningful and visually distinctive. However, a dedicated benchmark for bus front-view object detection remains largely absent.

Compared with existing datasets, bus front-view scenes exhibit several distinct visual characteristics. First, the elevated viewpoint shifts the scale distribution toward smaller distant vulnerable road users while leaving nearby vehicles at much larger scales. Second, bus stops and intersections frequently involve dense interactions among pedestrians, cyclists, and vehicles, resulting in heterogeneous and dynamic occlusions. Third, the observed object composition differs from that of conventional private-car datasets, with denser pedestrian regions and more complex stop-and-go interactions. These differences indicate a benchmark gap for scene-specific detection research. BFTD is constructed to fill this gap by providing a dedicated benchmark for visually challenging bus front-view detection.

## 3. Proposed Method

### 3.1. Bus Front-View Traffic Dataset

As one of the core contributions of this work, we introduce the Bus Front-View Traffic Dataset (BFTD), which is constructed for object detection in visually challenging bus front-view scenes. Unlike conventional private-car datasets, BFTD is collected from bus-mounted forward-facing cameras with an elevated viewpoint. This viewpoint provides a broader observable region while inducing a distinct scale distribution and scene layout. In particular, vulnerable road users such as pedestrians and cyclists frequently appear as small or partially occluded targets in distant regions, whereas nearby vehicles occupy medium- and large-scale regions. These characteristics make BFTD a representative benchmark for studying scene-specific object detection under severe scale variation and heterogeneous occlusion.

### 3.2. Data Collection and Annotation

BFTD is constructed from real-world traffic videos collected in Yantai, China. The videos were recorded on 10 October 2022 from forward-facing cameras mounted on multiple buses operating on multiple urban routes. The cameras were installed near the center of the windshield, forming a forward-looking elevated viewpoint. Compared with conventional low-view front-view settings, this configuration provides a wider field of view and captures more complex scene composition, including dense pedestrian regions, mixed motorized and non-motorized traffic, frequent partial occlusion, and substantial variation in illumination and weather. Frames were extracted at a resolution of 1920×1080 to preserve sufficient detail for small-object analysis. The recordings cover multiple time periods and include bus stops, intersections, and arterial roads.

The dataset includes five object categories: car, van, truck, pedestrian, and cyclist. These categories reflect the real object composition commonly observed in bus front-view scenes. In particular, the larger number of cars relative to other vehicle types reflects the actual scene distribution rather than a sampling bias introduced during curation. At the same time, pedestrians and cyclists are explicitly retained because they form visually challenging and safety-relevant small-object categories in elevated-view scenes.

To ensure annotation quality, BFTD adopts a semi-automatic annotation pipeline with multi-stage verification. Grounding DINO [34] is first used to generate preliminary bounding boxes. The initial predictions are then manually reviewed and refined by trained annotators through box correction, missing-object completion, and false-positive removal. Each image is checked independently by two annotators, and any discrepancies are resolved by discussion and final confirmation from a senior reviewer. In addition, approximately 10% of the dataset is randomly re-evaluated for quality control. Environmental-condition tags, including lighting and weather, are also assigned to facilitate condition-aware analysis.

By combining an elevated bus-mounted viewpoint with a rigorously verified annotation process, BFTD provides a scene-specific benchmark for evaluating object detection under scale variation, crowding, and partial occlusion.

### 3.3. Data Statistics

BFTD consists of high-resolution images captured using bus-mounted forward-facing cameras, with annotations provided in the YOLO format. The current release adopts predefined training, validation, and test subsets at a ratio of 7:2:1, ensuring that all compared detectors are evaluated under a consistent experimental protocol. Because the images are extracted from videos recorded by bus-mounted cameras, a purely image-level split may not completely eliminate temporal or scene-level correlations. Therefore, we do not interpret the current split as providing a fully sequence-independent evaluation. Instead, sequence-aware partitioning based on routes, trips, or temporally adjacent video segments is regarded as an important refinement for future releases of BFTD. The final dataset comprises 8131 images and 56,137 annotated instances across five categories—car (41,425), van (6796), truck (763), pedestrian (4279), and cyclist (2874)—reflecting substantial variation in object scale, category distribution, and environmental conditions.

**Figure 2:**
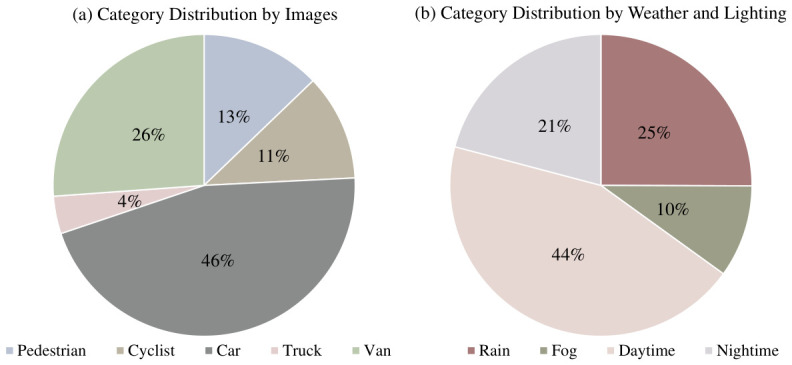
Image-level category-presence and environmental-condition distributions of the BFTD.

**Figure 3:**
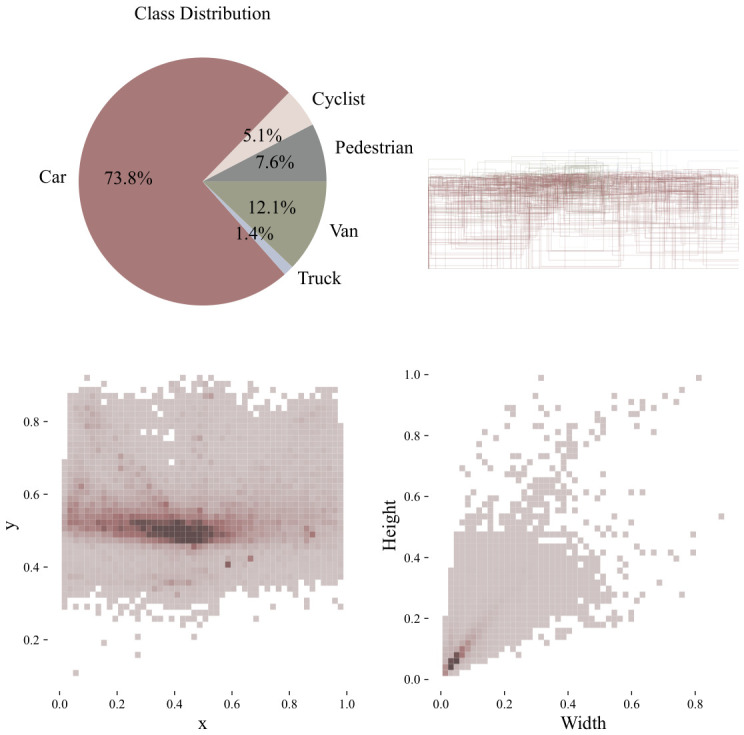
Instance-level annotation statistics of the BFTD. From the top left to the bottom right, the four subfigures represent the following: the distribution of annotated object instances by category, the anchor box distribution after k-means clustering, the distribution of bounding-box centers (x,y), and the distribution of bounding-box width and height (w,h).

It should be noted that Figure 2 and Figure 3 summarize BFTD from different statistical perspectives. Figure 2a reports image-level category presence, where an image is counted if at least one object of a given category appears, whereas Figure 3 reports instance-level annotation statistics based on the number of annotated bounding boxes for each category. Therefore, the category proportions in the two figures are not expected to be identical. The condition distribution in Figure 2b further shows that daytime scenes form the majority, complemented by nighttime, rainy, and foggy scenes, providing environmental diversity for robustness evaluation.

Compared with widely used benchmarks such as KITTI, Cityscapes, and BDD100K, BFTD provides a complementary bus-mounted front-view perspective rather than claiming to be universally more difficult under every statistic. Its value lies in capturing elevated camera geometry, bus-stop and intersection interactions, and traffic-participant composition that are not fully represented by conventional private-car or roadside benchmarks. The combination of high-resolution imagery (1920×1080), a large number of annotated traffic-participant instances, and diverse visual conditions makes BFTD a useful scene-specific benchmark for bus-mounted visual sensing.

Figure 3 further visualizes category-level instance statistics, k-means anchor distributions, and the spatial distributions of bounding-box centers and scales. The results indicate substantial diversity in both object size and spatial layout: larger objects are concentrated mainly in the lower-middle region of the image, while smaller objects are more frequently distributed in central and distant regions, often under crowded or partially occluded conditions. These characteristics highlight the need for both fine-grained small-object representation and robust contextual modeling.

**Figure 4:**
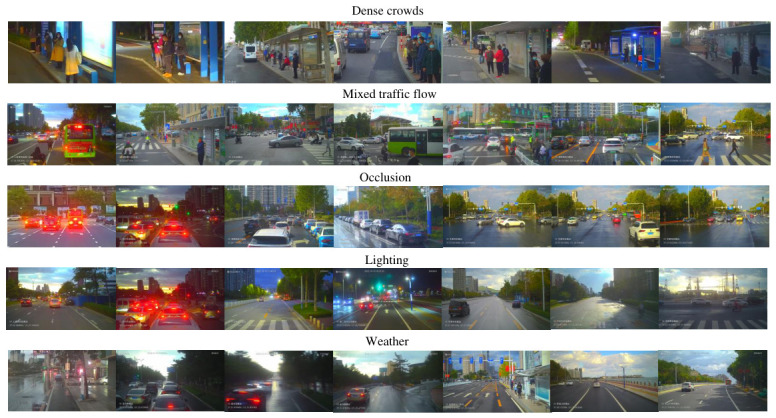
Representative examples from the BFTD under diverse scenarios. The dataset covers visually challenging traffic scenes including dense crowds, mixed-traffic flow, occlusion, illumination variation, and different weather conditions.

Representative examples in Figure 4 further illustrate the visual complexity of BFTD.

### 3.4. Quantitative Comparison of Visual Properties

To more carefully characterize the visual properties of bus-mounted front-view sensing, we compare BFTD with representative public traffic datasets from the perspectives of object scale, object density, and potential occlusion. This comparison is not intended to claim that BFTD is universally more difficult than all existing benchmarks; rather, it identifies the different visual profile introduced by the elevated bus-mounted viewpoint and traffic-participant composition. Table 2 reports annotation-derived statistics computed from the released bounding-box annotations using the same definitions across all datasets. Objects/Image denotes the average number of annotated objects per image. The small-object ratio is defined as the proportion of boxes whose normalized area is smaller than 0.0025, corresponding to the COCO small-object threshold of 32×32 pixels under a 640×640 input resolution. The mean box area ratio is the average bounding-box area divided by the image area. Since explicit visible-region occlusion labels are not consistently available across datasets, we report an overlap-derived occlusion proxy, where an instance is counted as overlap-affected if $O_{\max}\geq 0.10$, with $O_{\max}$ defined as the maximum intersection area with any other box in the same image divided by the area of the instance itself.

These statistics indicate that BFTD differs from conventional traffic datasets in object scale, density, and overlap tendency, but they also show that the challenge profile is not identical across benchmarks. For example, BDD100K has higher object density, a higher small-object ratio, and a higher overlap-derived proxy than BFTD, partly because its annotations include traffic signs and traffic lights in addition to traffic participants. Therefore, the contribution of BFTD should not be interpreted as providing the most crowded or universally most difficult dataset. Instead, BFTD complements existing benchmarks by focusing on bus-mounted, elevated front-view traffic-object sensing, where distant vulnerable road users, bus-stop interactions, and mixed-traffic composition form a distinct perception setting. The mean box area ratio of BFTD remains lower than that of KITTI, supporting the presence of many small or distant targets under the elevated bus-mounted viewpoint, while the overlap-derived proxy indicates that object interaction and partial overlap are still common in BFTD.

### 3.5. Overall Architecture

Guided by the structural properties of bus front-view scenes discussed in previous sections, we design YOLO-M2LA as a mechanism-oriented object detection framework built upon YOLOv9. YOLOv9 is selected as the base architecture because it provides a stable and reproducible programmable gradient information design, competitive accuracy–efficiency trade-off, and a modular backbone–neck–head structure that allows controlled replacement of downsampling and neck components. Newer YOLO variants are used as comparison baselines rather than as the modification backbone, so that the effect of the proposed CBS-SPD and M2LA modules can be isolated without confounding differences from different base architectures. Although YOLO-based detectors provide strong efficiency and competitive accuracy, they still face two limitations in this scene-specific setting. First, small and distant targets may lose critical fine-grained cues during early downsampling. Second, conventional convolutional neck structures are often insufficient for resolving heterogeneous occlusion in dense mixed-traffic scenes.

To address these issues, YOLO-M2LA introduces two targeted components. In the backbone, conventional stride-based downsampling is replaced by the CBS-SPD module, which performs information-preserving downsampling and retains spatial details useful for small-object detection under elevated-view conditions. In the neck, the original RepNCSPELAN4 module in YOLOv9 is replaced by the proposed M2LA block, which serially combines Multi-Scale Dilated Attention (MSDA) and Mamba-inspired Linear Attention (MLLA). This design enables a balanced integration of local multi-scale reasoning and efficient global contextual aggregation for target disambiguation under occlusion.

**Figure 5:**
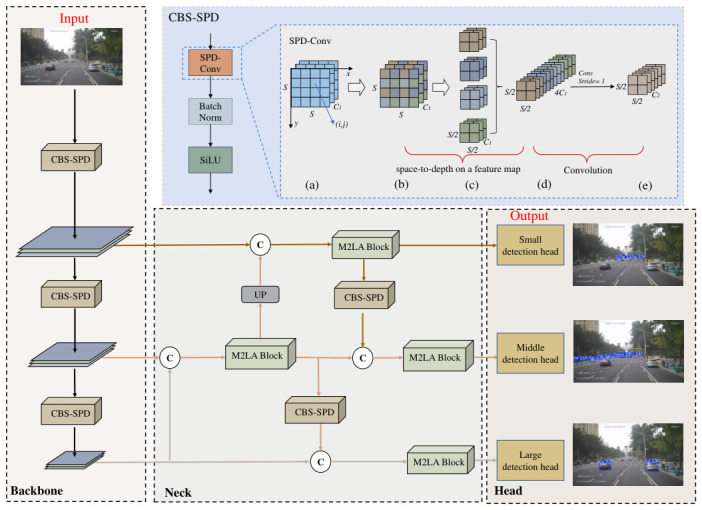
Overall architecture of the proposed YOLO-M2LA framework and the CBS-SPD module. Subfigure (**a**) shows the input feature map, subfigures (**b**–**d**) illustrate the space-to-depth rearrangement process, and subfigure (**e**) shows the output feature map after convolution.

As illustrated in Figure 5, YOLO-M2LA integrates CBS-SPD into the backbone and M2LA into the neck, while retaining three detection heads for small, medium, and large objects. In this way, the framework remains compatible with multi-scale prediction while being explicitly adapted to the severe scale variation and occlusion complexity of bus front-view scenes.

The purpose of the proposed design is not to claim each operator as an entirely new detection principle, but to integrate complementary mechanisms into YOLOv9 for the specific sensing challenges of bus-mounted front-view traffic perception. CBS-SPD is introduced for early-stage fine-detail preservation, MSDA is adapted for local multi-scale context modeling, and MLLA is used for efficient global dependency aggregation. Their combined role is to provide a scene-oriented architecture for small and occlusion-prone traffic participants under elevated-view sensing.

### 3.6. CBS-SPD for Fine-Grained Feature Preservation

In the backbone, we introduce the CBS-SPD module to replace conventional stride convolutions. The key idea is to combine a space-to-depth (SPD) transformation with a non-stride convolution [35], thereby performing information-preserving downsampling. Instead of directly discarding spatial details through stride-2 convolution, CBS-SPD reorganizes local spatial patterns into the channel dimension before convolutional processing, which helps preserve discriminative cues for small-object detection.

As illustrated in Figure 5, the transformation process of the SPD-based module is explained using scale=2 as an example. The input feature map has size $\left(S,S,C_{1}\right)$, as shown in Figure 5a. The SPD layer proceeds in four steps. First, the spatial domain of the input is divided into four sub-regions, as illustrated in Figure 5b. Second, each sub-region is downsampled to form a feature map of size $\left(\frac{S}{2},\frac{S}{2},C_{1}\right)$, as shown in Figure 5c. Third, these four feature maps are concatenated along the channel dimension, yielding a feature map of size $\left(\frac{S}{2},\frac{S}{2},4C_{1}\right)$, as shown in Figure 5d. Finally, a standard convolution with stride 1 is applied to produce the output feature map of size $\left(\frac{S}{2},\frac{S}{2},C_{2}\right)$, as shown in Figure 5e.

This design is particularly suitable for elevated-view front-view scenes. In distant regions, pedestrians and cyclists often occupy only a few pixels, and their discriminative details can be easily lost in conventional early downsampling. By remapping spatial information into channel dimensions before convolution, CBS-SPD better preserves fine-grained textures and edge structures. As a result, it improves the representation of small and partially occluded targets and strengthens recall under visually cluttered conditions.

### 3.7. M2LA for Local–Global Contextual Disambiguation

**Figure 6:**
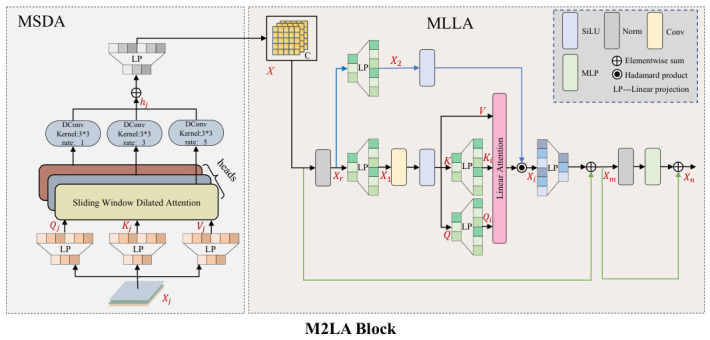
The architecture of the proposed M2LA block. The left part illustrates the MSDA (Multi-Scale Dilated Attention), which captures contextual features with dilation rates {1,3,5}, while the right part shows the MLLA (Mamba-inspired Linear Attention), which achieves efficient global aggregation with linear complexity. These two modules are connected in series to form M2LA.

In the neck of the detection network, we design the Multi-Scale and Mamba Linear Attention (M2LA) block to replace the RepNCSPELAN4 module in YOLOv9. As shown in Figure 6, M2LA adopts a serial architecture consisting of two complementary components: Multi-Scale Dilated Attention (MSDA), which emphasizes local multi-scale context modeling, and Mamba-inspired Linear Attention (MLLA), which performs efficient global dependency aggregation. This serial design improves target disambiguation in occlusion-prone bus front-view scenes without introducing excessive computational overhead. A serial structure is adopted instead of parallel fusion because the two stages have different functional roles: MSDA first enriches local multi-scale spatial evidence, and MLLA then aggregates broader dependencies on the enhanced representation. This sequential ordering avoids directly mixing unrefined local and global responses and keeps the block compatible with the hierarchical detection neck.

**Multi-Scale Dilated Attention (MSDA).** Given an input feature map *X*, a linear projection is first applied to generate the query (*Q*), key (*K*), and value (*V*). The channel dimension is then divided into *n* attention heads, each associated with a different dilation rate $r_{j}\in \left\{1,3,5\right\}$. For the *j*-th head, sliding-window dilated attention (SWDA) is performed as

(1) $$h_{j}=\mathrm{SWDA}\left(Q_{j},K_{j},V_{j},r_{j}\right),\;1\leq j\leq n,$$

where $Q_{j}$, $K_{j}$, and $V_{j}$ denote the corresponding channel partitions. The outputs of all heads are concatenated and linearly projected:

(2) $$X=\mathrm{Linear}(\mathrm{concat}\left[h_{1},\ldots ,h_{n}\right]).$$

The MSDA design is inspired by the Multi-Scale Dilated Attention mechanism introduced in DilateFormer [36], but it is adapted here to a YOLOv9 detection neck for dense traffic object detection rather than used as a transformer classification backbone. By assigning different dilation rates to different heads, MSDA enlarges the effective receptive field and captures contextual information at multiple local scales. This is particularly useful in bus front-view scenes, where small pedestrians and cyclists often appear in dense local neighborhoods and require stronger local context to be separated from cluttered backgrounds or adjacent objects. In our implementation, MSDA uses four attention heads, and pre-normalization is applied before attention computation to stabilize training.

**Mamba-inspired Linear Attention (MLLA).** While MSDA focuses on local multi-scale reasoning, robust detection in bus front-view scenes also requires global context to resolve ambiguities caused by heterogeneous occlusion. To this end, we introduce MLLA, which performs global feature aggregation with linear computational complexity.

Specifically, the output of MSDA, denoted by *X*, is fed into the MLLA module, where linear projections generate two feature branches, $X_{1}$ and $X_{2}$. The branch $X_{1}$ is further mapped into queries, keys, and values. Queries and keys are projected into *h* subspaces using distinct learnable projection matrices:

(3) $$Q_{i}=QW_{i}^{Q},\;K_{i}=KW_{i}^{K},$$

where $W_{i}^{Q}$ and $W_{i}^{K}$ denote the projection matrices of the *i*-th head. Linear attention is then computed as

(4) $$\mathrm{Attention}\left(Q_{i},K_{i},V\right)=\frac{\varphi \left(Q_{i}\right)\left(\varphi {\left(K_{i}\right)}^{⊤}V\right)}{\varphi \left(Q_{i}\right)\varphi {\left(K_{i}\right)}^{⊤}},$$

where φ(·) denotes the feature-map kernel used to ensure linear complexity. The resulting attention output is modulated by a gating mechanism derived from $X_{2}$:

(5) $$X_{i}=\mathrm{Attention}\left(Q_{i},K_{i},V\right)⊙\mathrm{SiLU}\left(X_{2}\right).$$

The gated features are linearly transformed and combined with a residual connection:

(6) $$X_{m}=X+\mathrm{Linear}\left(X_{i}\right),$$

followed by normalization and a multilayer perceptron (MLP):

(7) $$X_{n}=X_{m}+\mathrm{MLP}\left(\mathrm{Norm}\left(X_{m}\right)\right).$$

**Implementation Details and Stability.** In MLLA, we employ four attention heads and apply Layer Normalization in a pre-normalization manner before both MSDA and MLLA. Residual connections are used throughout the block to facilitate gradient propagation. A dropout rate of 0.1 is applied after attention projection to improve training stability. To control computational overhead, all attention operations are performed on channel-reduced feature representations.

By serially combining MSDA and MLLA, M2LA achieves a balanced integration of local multi-scale reasoning and efficient global contextual aggregation. This design is particularly suitable for bus front-view scenes, where local appearance details alone are often insufficient under dense mixed traffic and frequent partial occlusion. Thus, M2LA complements CBS-SPD by improving context-aware feature aggregation in the neck.

## 4. Experimental Section

To comprehensively evaluate YOLO-M2LA, we conduct experiments on the BFTD dataset together with several public benchmarks. The evaluation is organized from four complementary perspectives: comparison with representative detectors, model complexity analysis, robustness under diverse visual conditions, and mechanism verification through ablation, cross-dataset transfer, and qualitative analysis. Unless otherwise stated, all experiments are conducted on the publicly released YOLO-format BFTD dataset with fixed training, validation, and testing splits. The purpose of this section is not only to report quantitative performance, but also to analyze how the proposed design responds to the scene-specific challenges of bus front-view detection.

### 4.1. Experimental Setup

**Evaluation metrics.** We adopt COCO-style evaluation metrics, which jointly measure precision and recall under multiple IoU thresholds and object scales. Specifically, we report mAP@0.5, mAP@0.5:0.95, ${\mathrm{AP}}_{S}$, ${\mathrm{AP}}_{M}$, ${\mathrm{AP}}_{L}$, AR@100, ${\mathrm{AR}}_{S}$, ${\mathrm{AR}}_{M}$, and ${\mathrm{AR}}_{L}$. Among these metrics, ${\mathrm{AP}}_{S}$ and ${\mathrm{AR}}_{S}$ are especially important because they directly reflect the ability to detect and recall small targets, which frequently appear in distant regions of bus front-view scenes.

**Training protocol.** To provide a controlled comparison, all detectors are trained under a unified experimental protocol. Input images are resized to 640×640. Data augmentation includes random adjustment of hue, saturation, and brightness in HSV space, random erasing with probability 0.4, and horizontal flipping with probability 0.5. All models are trained for 100 epochs using stochastic gradient descent (SGD), with batch size 32, initial learning rate 0.01, and weight decay 0.0005. The learning rate is decayed by a factor of 10 every 20 epochs. All implementations are based on PyTorch.

**Hardware environment and model variants.** All experiments were implemented in PyTorch 2.5.0 with CUDA 11.8 acceleration. To ensure a fair comparison, all compared models were trained and evaluated under the same software and hardware environment. Inference latency was measured on an NVIDIA RTX 3090 GPU using the same input resolution and batch size. Following the YOLO model-scaling convention, YOLO-M2LA-n and YOLO-M2LA-x denote the lightweight and large-capacity variants, respectively. They share the same proposed CBS-SPD and M2LA designs, while differing in the depth and width scaling of the backbone, neck, and detection head. Unless otherwise specified, ablation and cross-dataset analyses are conducted using YOLO-M2LA-n for fair comparison with lightweight baselines, whereas per-class evaluation reports YOLO-M2LA-x to analyze the best-performing model.

**Controlled comparison setting.** Certain detector families, especially transformer-based detectors, may benefit from task-specific optimization settings such as AdamW, longer training schedules, or customized augmentations. In this work, however, we intentionally adopt a unified protocol to isolate architectural differences under the same training budget and preprocessing setting. Therefore, the results should be interpreted as a controlled comparison rather than the individually optimized upper bound of each baseline.

### 4.2. Comparison with Representative Detectors

We compare YOLO-M2LA with representative detectors from three main families: two-stage detectors (Faster R-CNN and Cascade R-CNN), one-stage detectors (SSD, RetinaNet, YOLOv5, YOLOv6, YOLOv8, YOLOv9, YOLOv10, and YOLO11), and transformer-based detectors (Deformable DETR, F-DETR, and DINO). The quantitative results are summarized in Table 3.

Under the unified protocol, YOLO-M2LA achieves consistent improvements over strong baselines at both lightweight and large model scales. Compared with classical two-stage detectors, YOLO-M2LA-n improves mAP@0.5 by +0.095 over Faster R-CNN and +0.123 over SSD, while also providing clear gains on small-object metrics. Relative to representative one-stage baselines such as YOLOv6-n and YOLOv9-n, YOLO-M2LA-n achieves higher mAP@0.5:0.95 together with improved ${\mathrm{AP}}_{S}$ and ${\mathrm{AR}}_{S}$, indicating enhanced sensitivity to small and visually crowded targets. Similar trends can also be observed at the large scale, where YOLO-M2LA-x outperforms all compared YOLO baselines in both overall detection accuracy and scale-aware metrics.

When compared with transformer-based detectors, YOLO-M2LA also performs favorably under the same training budget. For instance, YOLO-M2LA-n achieves higher mAP@0.5:0.95 than Deformable DETR, F-DETR, and DINO in the controlled setting. This result suggests that the proposed mechanism-oriented design effectively strengthens both fine-grained feature representation and contextual reasoning without relying on the higher training cost typically associated with transformer detectors.

The gains are especially evident on ${\mathrm{AP}}_{S}$ and ${\mathrm{AR}}_{S}$. On BFTD, small targets often correspond to distant pedestrians and cyclists located near bus stops or intersections. The superior small-object performance of YOLO-M2LA indicates that the proposed CBS-SPD and M2LA modules effectively address the fine-detail loss and contextual ambiguity that commonly arise in bus front-view scenes. Overall, the results in Table 3 show that YOLO-M2LA consistently improves scene-specific object detection quality across different model scales.

Although BFTD does not provide explicit occlusion labels, the consistent improvement in small-object metrics together with qualitative crowded-scene results suggests that YOLO-M2LA is more robust in visually challenging conditions where object overlap and partial occlusion are frequent.

### 4.3. Per-Class and Overlap-Level Analysis

Because BFTD is naturally imbalanced, with cars forming the majority of instances and pedestrians, cyclists, and trucks appearing less frequently, per-class evaluation is necessary to determine whether the proposed detector improves only dominant classes or also benefits safety-relevant minority classes. Table 4 reports the class-wise detection performance of YOLO-M2LA-x on the held-out BFTD evaluation split used for the main comparison in Table 3. The image count denotes the number of evaluation images containing at least one instance of the corresponding class, while the instance count denotes the number of ground-truth objects of that class. Since one image may contain multiple object categories, the class-wise image counts are not mutually exclusive.

To characterize occlusion-prone cases without relying on unavailable visible-region annotations, we further compute an overlap-derived proxy from the bounding-box annotations. For each instance, $O_{\max}$ is defined as the maximum intersection area between this instance and any other annotated box in the same image, normalized by the area of the instance itself. Instances are divided into four overlap levels: non-overlapped ($O_{\max}<0.10$), slight overlap ($0.10\leq O_{\max}<0.30$), moderate overlap ($0.30\leq O_{\max}<0.50$), and heavy overlap ($O_{\max}\geq 0.50$). This proxy should not be interpreted as a manually labeled physical occlusion ratio, but it provides a reproducible annotation-derived measure of object crowding and potential occlusion.

Table 5 reports the overlap-level statistics which show that 25.92% of the annotated instances are affected by at least slight box overlap, and 20.80% fall into moderate or heavy overlap levels. These results do not constitute manually labeled physical occlusion rates, but they quantitatively support the observation that BFTD contains frequent object crowding and potential occlusion, especially in mixed-traffic scenes around bus stops and intersections.

### 4.4. Model Complexity Analysis

In addition to detection accuracy, we analyze the complexity and inference efficiency of YOLO-M2LA. Table 6 reports the number of parameters, FLOPs, inference latency, and mAP@0.5:0.95 for representative YOLO-based models. Inference latency is measured with batch size 1 under FP32 precision on an NVIDIA RTX 3090 GPU. FPS is also reported for direct deployment interpretation. Since no embedded-platform experiment has yet been conducted, we do not claim direct real-time embedded deployment; instead, embedded deployment and vibration/blur robustness are discussed as limitations and future work.

Since YOLO-M2LA is developed based on YOLOv9, the most direct comparison is with YOLOv9 variants. Compared with YOLOv9-n, YOLO-M2LA-n increases the parameter count only modestly from 1.73 M to 1.93 M and changes latency from 14.8 ms to 16.2 ms, while improving mAP@0.5:0.95 from 0.536 to 0.542. The FLOPs remain nearly unchanged, suggesting that the performance improvement is mainly produced by better fine-grained feature preservation and contextual modeling rather than substantially increased computation.

A similar trend appears at the larger model scale. YOLO-M2LA-x achieves 0.632 mAP@0.5:0.95, which is 0.009 higher than YOLOv9-x, with only moderate increases in latency and comparable complexity. These results indicate that the proposed CBS-SPD and M2LA modules provide a favorable balance between visual representation enhancement and computational overhead.

### 4.5. Robustness Under Diverse Visual Conditions

To further evaluate robustness under different visual conditions, we conduct experiments on four representative BFTD subsets: daytime, nighttime, rain, and fog. The corresponding subsets contain 3585, 1695, 2034, and 817 images, respectively. Each subset is divided into training, validation, and testing sets using a 7:2:1 ratio. In addition to condition-specific models, we train a unified YOLO-M2LA-n model on the full dataset to study the effect of joint multi-condition learning. The results are reported in Table 7.

The unified model achieves the highest overall performance, reaching 0.816 mAP@0.5 and 0.542 mAP@0.5:0.95, which exceeds all condition-specific models. This indicates that joint training across heterogeneous visual conditions improves feature generalization and reduces domain bias. Among the single-condition models, daytime training yields the strongest results because of the relatively abundant high-visibility data, whereas nighttime and fog performance degrades because of low contrast, reduced visibility, and noisier visual cues. Rainy-condition performance remains intermediate, suggesting that YOLO-M2LA maintains reasonable robustness even under reflection interference and partial visibility degradation.

From a scale-aware perspective, unified training also produces more balanced performance across ${\mathrm{AP}}_{S}$, ${\mathrm{AP}}_{M}$, and ${\mathrm{AP}}_{L}$. In particular, the unified model preserves stronger small-object recall than several adverse-condition models, indicating that diverse-condition learning helps maintain fine-grained cues under more visually difficult environments.

### 4.6. Ablation Study

To isolate the contribution of each proposed component, we conduct ablation experiments on top of the YOLOv9 baseline. The unmodified YOLOv9-n baseline is included in the same table, followed by four configurations: +CBS-SPD, which replaces stride convolutions in the backbone with information-preserving downsampling; +MSDA, which introduces local multi-scale contextual modeling; +MLLA, which introduces efficient global aggregation; and the full YOLO-M2LA-n model, which combines CBS-SPD with MSDA and MLLA. The results are summarized in Table 8.

Compared with the YOLOv9-n baseline, each component contributes differently to the final gain. CBS-SPD provides the strongest improvement on small-object metrics, increasing ${\mathrm{AP}}_{S}$ to 0.101 and ${\mathrm{AR}}_{S}$ to 0.192. This supports the claim that preserving fine-grained spatial cues during early downsampling is critical for distant small-object perception in bus front-view scenes. MSDA yields the largest improvement in mAP@0.5:0.95 among the individual attention components, indicating its effectiveness in strengthening local multi-scale representation. MLLA further improves overall recall, suggesting that efficient global contextual aggregation helps stabilize detection under clutter and partial occlusion.

The full YOLO-M2LA-n model achieves the best results across all reported metrics, with 0.816 mAP@0.5, 0.542 mAP@0.5:0.95, and 0.115 ${\mathrm{AP}}_{S}$. These results show that CBS-SPD and M2LA are highly complementary: the former preserves fine-grained spatial detail, while the latter strengthens contextual disambiguation. Therefore, the ablation study supports the mechanism-oriented design rationale introduced in the Section 3.

### 4.7. Supplementary Cross-Dataset Transfer Evaluation

To obtain a preliminary assessment of cross-dataset robustness, we evaluate YOLO-M2LA-n in a zero-shot setting, where the model is trained exclusively on BFTD and directly tested on external public benchmarks without fine-tuning or domain adaptation. We consider three traffic datasets: Cityscapes, BDD100K, and KITTI. These datasets contain realistic urban scenes but differ substantially from BFTD in viewpoint, annotation style, and class taxonomy.

To make the evaluation as fair as possible, a consistent category mapping is applied. Specifically, the person class in Cityscapes and BDD100K is mapped to the *pedestrian* category in BFTD, while corresponding vehicle categories such as *car* and *truck* are aligned whenever possible. Classes without a clear correspondence are excluded from evaluation, and predictions assigned to unmapped classes are ignored when computing the metrics.

As reported in Table 9, YOLO-M2LA-n achieves higher evaluation scores than the compared baselines across the three datasets under this zero-shot setting. The gains are especially visible on ${\mathrm{AP}}_{S}$, where YOLO-M2LA-n exceeds the strongest baseline by +0.029 on Cityscapes, +0.030 on BDD100K, and +0.026 on KITTI. These results suggest that the proposed combination of fine-grained feature preservation and local–global contextual modeling transfers reasonably well to unseen urban scenes. At the same time, this experiment should be interpreted as supplementary robustness evidence rather than a substitute for target-domain training.

### 4.8. Qualitative Results and Failure Analysis

To further analyze the behavior of YOLO-M2LA, we present qualitative evidence from three perspectives: visual interpretability, direct detection comparison, and representative result inspection.

**Figure 7:**
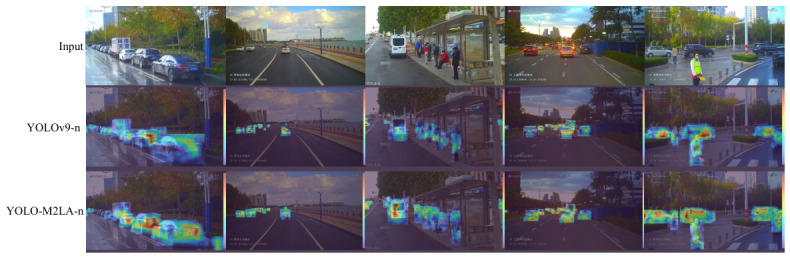
Visualization of Class Activation Maps (Grad-CAM++) comparing YOLOv9-n and the proposed YOLO-M2LA-n on vehicle detection.

**Visual interpretability.** We adopt Class Activation Maps (CAMs) [42] and specifically Grad-CAM++ [43] to visualize the spatial regions contributing to the detector’s decisions. As shown in Figure 7, YOLO-M2LA-n exhibits stronger and more concentrated activation around object contours and discriminative regions than YOLOv9-n, while YOLOv9-n tends to spread attention over less relevant background areas. This observation supports the claim that the proposed framework captures both fine-grained detail and meaningful contextual cues more effectively.

**Figure 8:**
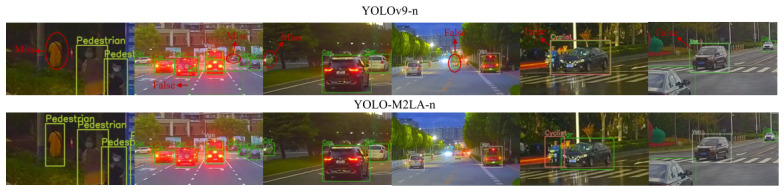
Detection differences between YOLOv9-n and YOLO-M2LA-n.

**Figure 9:**
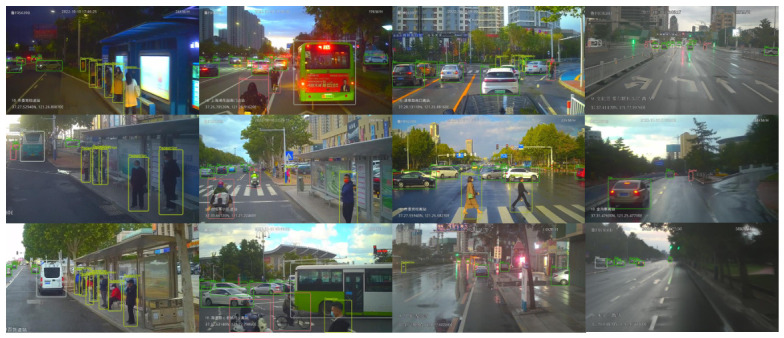
Representative detection results of YOLO-M2LA-x on the BFTD dataset. The model achieves accurate recognition of small pedestrians, riders, and vehicles under dense traffic, frequent occlusion, and varying illumination conditions.

**Qualitative comparison.** Figure 8 compares YOLO-M2LA-n and YOLOv9-n in visually challenging bus front-view scenes with dense pedestrians, mixed traffic, and frequent occlusion. Local enlarged views show that YOLO-M2LA-n detects more small-scale pedestrians and riders that may be missed or misclassified by YOLOv9-n. Figure 9 further illustrates representative BFTD results, where YOLO-M2LA-x successfully recognizes vulnerable road users and vehicles under crowding, congestion, complex overlap, and illumination variation. These observations are consistent with the quantitative improvements reported in Table 3.

**Figure 10:**
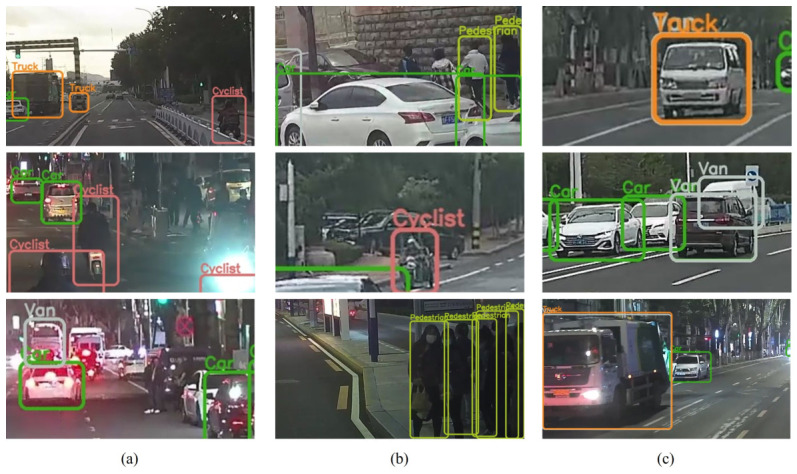
Representative failure cases of YOLO-M2LA-x on BFTD: (**a**) missed detections under adverse visibility, (**b**) instance confusion under heavy occlusion and crowding, and (**c**) misclassification between visually similar vehicle subclasses.

**Failure cases and limitations.** Despite the overall improvement in YOLO-M2LA-n and YOLO-M2LA-x, failure cases still occur in highly challenging bus front-view scenes, as illustrated in Figure 10. These failures mainly arise under adverse visibility, heavy occlusion and crowding, and ambiguous appearance between similar vehicle subclasses. In such conditions, distant pedestrians, cyclists, or small vehicles may provide only weak visual evidence, adjacent instances may be partially merged or suppressed in dense mixed traffic, and visually similar categories such as *van* and *truck* may occasionally be confused. These observations indicate that severe visibility degradation, extreme crowding, and fine-grained class ambiguity remain open challenges for bus front-view detection. Future work may benefit from more explicit occlusion-aware supervision, stronger adaptation to adverse weather and nighttime conditions, and more discriminative feature learning for visually similar subclasses.

## 5. Limitations and Future Work

Several limitations should be acknowledged. First, BFTD is collected from one city and one collection date. Although the dataset covers multiple bus routes, lighting conditions, rain, fog, bus stops, intersections, and arterial roads, geographic and temporal diversity remains limited. Future extensions should include additional cities, seasons, camera configurations, and traffic cultures. Second, the current dataset does not provide manual visible-region or occlusion-ratio annotations. Therefore, the overlap-level analysis in Table 5 is based on an overlap-derived proxy and should be interpreted as a reproducible approximation rather than a substitute for explicit human-labeled occlusion levels. Third, although the annotation process includes independent checking by two annotators and senior review, formal inter-annotator agreement statistics are not reported in the current release. Fourth, camera vibration and motion blur are important factors for bus-mounted visual sensing, especially under uneven road conditions, but they are not separately annotated in the current version. Fifth, the current efficiency analysis is measured on a desktop GPU, and embedded-device latency, memory footprint, and energy consumption should be evaluated before claiming direct deployment on edge platforms. Finally, the cross-dataset experiment is intended as supplementary zero-shot evidence; differences in taxonomy, annotation policies, and viewpoint geometry mean that it should not be interpreted as a fully optimized target-domain benchmark. In addition, because the current split is not presented as a fully sequence-independent protocol, possible temporal or scene-level correlations among images extracted from the same bus videos should be further reduced through a route- or sequence-aware split in a future release. In addition, BFTD exhibits a natural class imbalance, with cars accounting for the majority of instances and trucks appearing much less frequently. Although the reported class-wise AP indicates that YOLO-M2LA can still detect minority classes, the present study does not explicitly investigate balanced sampling, class-aware loss design, calibration, or uncertainty modeling for minority categories. These limitations define clear directions for future dataset expansion, annotation refinement, imbalance-aware learning, and deployment-oriented evaluation.

## 6. Conclusions

This paper presented BFTD, a high-resolution benchmark for bus front-view traffic object detection, and YOLO-M2LA, a local–global attention detector designed for small and occlusion-prone traffic participants. By combining CBS-SPD for information-preserving downsampling with M2LA for multi-scale and global context aggregation, the proposed framework improves detection performance on BFTD while maintaining practical computational complexity. Dataset statistics, per-class analysis, overlap-level statistics, ablation studies, and qualitative results jointly verify the relevance of the proposed design under challenging bus-mounted front-view conditions. Future work will extend BFTD to broader geographic and temporal settings, introduce more explicit occlusion and motion-degradation annotations, and investigate video-based temporal modeling for more robust public transportation perception.

## Figures and Tables

**Table 1:** Comparison of BFTD with representative traffic perception datasets from the perspective of viewpoint, task focus, and relevance to bus front-view detection.

Dataset	Platform	Task	Object	Limitations
KITTI [21]	Low-view	2D/3D detection	Vehicles	Mainly suburban scenes
Cityscapes [22]	Low-view	Semantic segmentation	Pedestrians, bicycles	Limited object detection focus; insufficient bus-front-view characteristics
BDD100K [23]	Low-view	2D detection	Diverse	Few dense bus-stop interactions; limited severe occlusion emphasis
nuScenes [24]	Multi-modal	3D perception	Diverse	LiDAR-oriented; 2D small-object analysis is limited
Waymo Open [25]	Low-view	Autonomous perception	Diverse	Large-scale but expensive in computation and storage
ApolloScape [26]	Low-view	Semantic segmentation/3D perception	Small objects	Focuses more on lanes and vehicles than mixed bus-front-view scenes
Argoverse [27]	Multi-modal	Trajectory prediction and perception	Vehicles	More oriented to motion forecasting than dense small-object detection
Caltech Ped. [28]	Low-view	Pedestrian detection	Pedestrians	Lacks vehicle and mixed-traffic composition
MOT Challenge [33]	Multi-view	Multi-object tracking	Pedestrians	Tracking-oriented rather than detection-oriented
CrowdHuman [29]	Low-view	Crowd detection	Pedestrians	Non-traffic scenarios
VisDrone [30]	Bird’s-eye	Small-object detection	Small objects	Aerial view differs substantially from vehicle-mounted perception
UA-DETRAC [31]	High-view	Vehicle detection and tracking	Vehicles	Static roadside cameras; lacks dynamic bus-mounted viewpoint
**BFTD (Ours)**	High-view	Traffic object detection	Dense pedestrians, non-motor, vehicles	Dedicated to bus front-view scenes with dense small objects and frequent occlusions

Note: Bold indicates the proposed dataset.

**Table 2:** Annotation-derived comparison of visual properties between BFTD and representative traffic datasets.

Dataset	Images	Objects/Image	Small-Object Ratio	Mean Box Area Ratio	Overlap-Derived Occlusion Proxy
KITTI	7481	5.42	20.08%	2.3889%	56.75%
BDD100K	10,000	12.37	60.28%	1.0381%	57.80%
BFTD	8131	6.90	26.81%	1.5995%	46.73%

**Table 3:** Comparison of YOLO-M2LA with representative object detectors on the BFTD dataset.

Method	mAP@0.5	mAP@0.5:0.95	AP_*S*_	AP_*M*_	AP_*L*_	AR@100	AR_*S*_	AR_*M*_	AR_*L*_
Faster R-CNN [3]	0.721	0.432	0.045	0.318	0.566	0.601	0.082	0.515	0.668
Cascade R-CNN [4]	0.748	0.451	0.051	0.336	0.583	0.619	0.094	0.534	0.681
SSD [5]	0.693	0.408	0.038	0.296	0.541	0.587	0.071	0.494	0.652
RetinaNet [6]	0.735	0.447	0.049	0.324	0.572	0.612	0.089	0.521	0.674
Deformable DETR [10]	0.762	0.483	0.072	0.351	0.591	0.641	0.146	0.542	0.689
F-DETR [20]	0.770	0.486	0.075	0.352	0.589	0.643	0.151	0.543	0.692
DINO [11]	0.781	0.495	0.079	0.367	0.603	0.652	0.158	0.553	0.698
YOLOv5-n [37]	0.790	0.527	0.071	0.391	0.609	0.662	0.140	0.593	0.717
YOLOv6-n [38]	0.780	0.518	0.053	0.385	0.597	0.659	0.110	0.594	0.712
YOLOv8-n [39]	0.799	0.534	0.067	0.415	0.606	0.667	0.162	0.606	0.716
YOLOv9-n [9]	0.797	0.536	0.088	0.404	0.613	0.668	0.172	0.603	0.719
YOLOv10-n [40]	0.718	0.516	0.071	0.391	0.591	0.669	0.206	0.606	0.718
YOLO11-n [41]	0.797	0.530	0.076	0.413	0.599	0.664	0.128	0.603	0.715
YOLO-M2LA-n (Ours)	**0.816**	**0.542**	**0.115**	**0.430**	**0.623**	**0.690**	**0.213**	**0.615**	**0.726**
YOLOv5-x [37]	0.871	0.612	0.135	0.504	0.665	0.708	0.287	0.653	0.753
YOLOv6-x [38]	0.868	0.603	0.115	0.490	0.660	0.690	0.202	0.634	0.735
YOLOv8-x [39]	0.876	0.617	0.124	0.517	0.665	0.703	0.247	0.648	0.746
YOLOv9-x [9]	0.880	0.623	0.160	0.524	0.669	0.708	0.322	0.658	0.748
YOLOv10-x [40]	0.865	0.601	0.148	0.486	0.653	0.696	0.284	0.645	0.736
YOLO11-x [41]	0.874	0.615	0.186	0.518	0.661	0.708	0.282	0.660	0.748
YOLO-M2LA-x (Ours)	**0.894**	**0.632**	**0.204**	**0.536**	**0.678**	**0.722**	**0.338**	**0.670**	**0.759**

Note: Bold indicates the best result in each column.

**Table 4:** Per-class detection performance of YOLO-M2LA-x on the held-out BFTD evaluation split.

Class	Eval. Images	Eval. Instances	AP@0.5	AP@0.5:0.95	Main Challenge
Car	1586	8244	0.960	0.750	Dominant class
Van	909	1348	0.910	0.718	Fine-grained vehicle confusion
Truck	125	136	0.799	0.575	Class imbalance
Pedestrian	434	871	0.821	0.459	Small size and occlusion
Cyclist	400	580	0.877	0.575	Small size and appearance variation

**Table 5:** Overlap-level statistics of BFTD using an overlap-derived proxy.

Overlap Level	Definition	Instances	Percentage
Non-overlapped	$O_{\max}<0.10$	29,905	53.27%
Slight overlap	$0.10\leq O_{\max}<0.30$	14,552	25.92%
Moderate overlap	$0.30\leq O_{\max}<0.50$	8152	14.52%
Heavy overlap	$O_{\max}\geq 0.50$	3528	6.28%

**Table 6:** Model complexity and inference efficiency comparison on the BFTD dataset. Latency is measured with batch size 1 under FP32 precision on an NVIDIA RTX 3090 GPU.

Model	Params (M)	FLOPs (G)	Latency (ms)	FPS	mAP@0.5:0.95
YOLOv8-n [39]	2.69	6.8	7.1	140.8	0.534
YOLOv9-n [9]	1.73	6.4	14.8	67.6	0.536
YOLO11-n [41]	2.58	6.3	8.4	119.0	0.530
**YOLO-M2LA-n (Ours)**	**1.93**	**6.5**	**16.2**	**61.7**	**0.542**
YOLOv8-x [39]	61.6	226.7	21.2	47.2	0.617
YOLOv9-x [9]	53.2	169.5	25.2	39.7	0.623
YOLO11-x [41]	56.8	194.4	21.0	47.6	0.615
**YOLO-M2LA-x (Ours)**	**55.3**	**178.2**	**27.4**	**36.5**	**0.632**

Note: Bold indicates the best result in each column.

**Table 7:** Condition-specific and baseline comparison of YOLO-M2LA-n on the BFTD dataset.

Test Condition	mAP@0.5	mAP@0.5:0.95	AP*_S_*	AP*_M_*	AP*_L_*	AR@100	AR*_S_*	AR*_M_*	AR*_L_*
Daytime	0.773	0.510	0.174	0.386	0.588	0.656	0.433	0.592	0.709
Nighttime	0.736	0.477	0.036	0.331	0.530	0.622	0.103	0.528	0.663
Rain	0.739	0.487	0.124	0.308	0.548	0.641	0.312	0.561	0.677
Fog	0.715	0.419	0.034	0.341	0.532	0.555	0.114	0.512	0.644
Unified	0.816	0.542	0.115	0.430	0.623	0.690	0.213	0.615	0.726

**Table 8:** Ablation study of YOLO-M2LA-n components on the BFTD dataset.

Test Condition	mAP@0.5	mAP@0.5:0.95	AP*_S_*	AP*_M_*	AP*_L_*	AR@100	AR*_S_*	AR*_M_*	AR*_L_*
YOLOv9-n	0.797	0.536	0.088	0.404	0.613	0.668	0.172	0.603	0.719
+CBS-SPD	0.803	0.531	0.101	0.417	0.614	0.678	0.192	0.604	0.717
+MSDA	0.808	0.539	0.097	0.421	0.617	0.681	0.186	0.609	0.721
+MLLA	0.810	0.534	0.095	0.419	0.618	0.686	0.187	0.611	0.724
YOLO-M2LA-n (Full)	**0.816**	**0.542**	**0.115**	**0.430**	**0.623**	**0.690**	**0.213**	**0.615**	**0.726**

Note: Bold indicates the best result in each column.

**Table 9:** Cross-dataset generalization results of YOLO-M2LA-n trained only on BFTD without fine-tuning.

Dataset	Method	mAP@0.5	mAP@0.5:0.95	AP*_S_*	AP*_M_*	AP*_L_*
Cityscapes	Faster R-CNN [3]	0.61	0.392	0.071	0.424	0.611
	YOLOv8-n [39]	0.64	0.408	0.084	0.436	0.623
	YOLOv9-n [9]	0.64	0.416	0.085	0.444	0.625
	Deformable DETR [10]	0.66	0.421	0.089	0.448	0.632
	YOLO-M2LA-n (Ours)	**0.69**	**0.445**	**0.118**	**0.467**	**0.648**
BDD100K	Faster R-CNN [3]	0.59	0.374	0.065	0.411	0.592
	YOLOv8-n [39]	0.62	0.391	0.078	0.429	0.604
	YOLOv9-n [9]	0.64	0.396	0.080	0.433	0.608
	Deformable DETR [10]	0.64	0.402	0.082	0.441	0.617
	YOLO-M2LA-n (Ours)	**0.67**	**0.426**	**0.112**	**0.459**	**0.633**
KITTI	Faster R-CNN [3]	0.71	0.452	0.092	0.482	0.664
	YOLOv8-n [39]	0.73	0.469	0.107	0.494	0.678
	YOLOv9-n [9]	0.72	0.472	0.109	0.498	0.679
	Deformable DETR [10]	0.75	0.482	0.111	0.507	0.689
	YOLO-M2LA-n (Ours)	**0.78**	**0.503**	**0.137**	**0.524**	**0.701**

Note: Bold indicates the best result in each column.

## Data Availability

The Bus Front-view Traffic Dataset (BFTD), annotation files, and implementation of YOLO-M2LA are publicly available at the project GitHub repository: https://github.com/motion-cv/BFTD-yolo-m2la (accessed on 3 August 2026). A DOI-based archival copy, such as Zenodo, will be considered in a future dataset release to further improve long-term accessibility.
